# Overexpression of the nucleocapsid protein of Middle East respiratory syndrome coronavirus up-regulates CXCL10

**DOI:** 10.1042/BSR20181059

**Published:** 2018-10-17

**Authors:** James Odame Aboagye, Chow Wenn Yew, Oi-Wing Ng, Vanessa M. Monteil, Ali Mirazimi, Yee-Joo Tan

**Affiliations:** 1 Institute of Molecular and Cell Biology, Agency for Science, Technology and Research (A*STAR), Singapore; 2 Department of Microbiology and Immunology, Yong Loo Lin School of Medicine, National University Health System (NUHS), National University of Singapore, Singapore; 3 Department for Laboratory Medicine, Karolinska Institute and Karolinska Hospital University, Solna, Sweden; 4 Public Health Agency of Sweden, Stockholm, Sweden

**Keywords:** CXCL10, Middle East Respiratory Syndrome coronavirus, nucleocapsid

## Abstract

Middle East respiratory syndrome coronavirus (MERS-CoV) causes respiratory diseases in humans and has a high mortality rate. During infection, MERS-CoV regulates several host cellular processes including antiviral response genes. In order to determine if the nucleocapsid protein of MERS-CoV (MERS-N) plays a role in viral–host interactions, a murine monoclonal antibody was generated so as to allow detection of the protein in infected cells as well as in overexpression system. Then, MERS-N was stably overexpressed in A549 cells, and a PCR array containing 84 genes was used to screen for genes transcriptionally regulated by it. Several up-regulated antiviral genes, namely *TNF, IL6, IL8*, and *CXCL10*, were selected for independent validation in transiently transfected 293FT cells. Out of these, the overexpression of MERS-N was found to up-regulate CXCL10 at both transcriptional and translational levels. Interestingly, CXCL10 has been reported to be up-regulated in MERS-CoV infected airway epithelial cells and lung fibroblast cells, as well as monocyte-derived macrophages and dendritic cells. High secretions and persistent increase of CXCL10 in MERS-CoV patients have been also associated with severity of disease. To our knowledge, this is the first report showing that the MERS-N protein is one of the contributing factors for CXCL10 up-regulation during infection. In addition, our results showed that a fragment consisting of residues 196–413 in MERS-N is sufficient to up-regulate CXCL10, while the N-terminal domain and serine-arginine (SR)-rich motif of MERS-N do not play a role in this up-regulation.

## Introduction

Middle East respiratory syndrome coronavirus (MERS-CoV) is a nidovirus and etiologic agent for respiratory disease. MERS-CoV emerged in Saudi Arabia in 2012 and is still causing respiratory infections with 2220 and 790 (35%) reported cases and deaths respectively [1]. Manifestations of MERS disease are similar to severe acute respiratory syndrome (SARS) with patients usually developing acute pneumonia that progresses to respiratory failure and acute respiratory disease syndrome [2]. Patients also exhibit extrapulmonary manifestations include renal failure, hepatic dysfunction, and diarrhoea with some severe cases of deranged coagulation profile and hematological changes [3–5].

MERS-CoV is an enveloped, single-stranded, positive-sense RNA virus, approximately 30 kb in length with two-thirds of its genome encoding 15–16 non-structural proteins (nsp) [6,7]. The remaining one-third encodes for structural proteins interspersed with accessory proteins. The structural proteins include the spike (S), matrix (M), envelope (E), and nucleocapsid (N) proteins [6,7]. For coronavirus, N primarily encapsidates the viral genome but also plays important roles in viral replication, virus particle assembly and release [8,9]. Additionally, the N protein of SARS-CoV has been reported to regulate host functions such as immune interference, apoptosis, proliferation, and cell cycle [10,11].

The N protein of MERS-CoV (to be referred to as MERS-N herein) is a 413 amino acids protein and has been reported to share some characteristics with N of other coronaviruses. For example, crystallography and small angle X-ray scattering experiment have shown that the N-terminal region of MERS-N exists as a monomer and has structural features that are similar to other coronavirus [12]. Like mouse hepatitis virus, porcine epidemic disease virus and SARS-CoV, MERS-N has been reported to be ADP-ribosylated [13] and acts as a viral suppressor of RNA silencing in mammalian cells [14]. However, the latter noted that MERS-N showed lower activity than the N proteins of other coronavirus tested. Similar to mouse hepatitis virus and SARS-CoV, MERS-N has been found to be essential for the packaging of viral RNA into virus-like particles [15].

During infection, the host defenses are activated to produce antivirals and proinflammatory cytokines and chemokines to help eliminate the infection. Interferon treatment has been reported to effectively subdue MERS-CoV replication [16]; however, like any successful viral infection, MERS-CoV has developed mechanisms to evade or dampen the activity of the host immune responses. Studies have demonstrated that MERS-CoV infection shows a delayed but marked induction of proinflammatory cytokines/chemokines [17]. In the present study, we aim to determine if the MERS-N protein is involved in regulating host antiviral response to infection. We showed that the MERS-N protein could contribute to the regulation of several host antiviral response genes including CXCL10. Importantly, up-regulation of CXCL10 has been reported in MERS patients [18,19] as well as MERS-CoV infected cells [3,17,20]. To our knowledge, this is the first time that MERS-N has been shown to be one of the contributing factors for CXCL10 up-regulation during infection and this suggests that MERS-N may contribute to viral pathogenesis.

## Materials and methods

### Cells culture and transfection

293FT cells (Invitrogen) were cultured in Dulbecco’s Modified Eagle’s Medium (DMEM, Hyclone), while A549 (American Type Culture Collection) was maintained in Minimal Essential Medium (Hyclone). All media were supplemented with 10% FBS (Hyclone) and 1% penicillin-streptomycin (Sigma–Aldrich). The *MERS-N* gene corresponding to the EMC-2012 strain was chemically synthesized (Genscript), while *N* genes of other coronaviruses were purchased (OriGene). All were subcloned into either pXJ40 or pXJ40-FLAG vector and used for transfection with Xtreme GENE^®^ (Sigma–Aldrich).

### Generation of mouse monoclonal antibody

The C-terminal fragment (residues 196–413) of MERS-N was fused to the glutathione S-transferase (GST) protein by cloning into the pGEX-6P1 vector (GE Healthcare). The protein was expressed in *Escherichia coli* and purified using GSH-sepharose beads (GE Healthcare). The GST-fusion protein was then used to immunize mice and generate hybridoma as previously described [21]. All mice were handled according to National Advisory Committee for Laboratory Animal Research (NACLAR) guidelines. Mouse monoclonal antibody (mAb) 7H6 was purified from the culture supernatant of a selected hybridoma by using a HiTrap Protein G column (GE Healthcare).

### Screening of binding sequence of mAb with peptides

Synthetic 15-mer peptides with ten amino acids overlapping sequences were generated (GL Biochem). Binding of the mAb to the peptides was screened by ELISA. Briefly, the plates were coated with peptides overnight, washed, and blocked for 30 min with 1% bovine albumin serum in 1× PBS. The mAb was then added and incubated at room temperature for 2 h. This was followed by washing, addition of secondary antibody goat anti-mouse HRP (Bio-Rad), and incubation at room temperature for 1 h. Plates were washed again, incubated with tetramethylbenzidine substrate (Pierce), and subsequently, the reaction was stopped with 2 M sulphuric acid. The absorbance was read at 450 nm.

### Generation of lentivirus

MERS-N was cloned into the pLenti6.3 vector (Thermo Fisher Scientific). 293FT cells were seeded at 3 × 10^6^ cells into 10-cm dishes and incubated at 37°C in 5% CO_2_ overnight. A plasmid mixture containing 2 µg each of pHDM-TatIb, pHDM-Hgpm2, pHDM-VSVG, and pPRb-CMV plasmids; and 8 µg of pLenti6.3-LacZ or pLenti6.3-MERS-N plasmid was prepared. The plasmid mixture was added to 500 µl of Opti-MEM (Gibco) and 16 µg of Xtreme GENE^®^. The mix was incubated at room temperature for 15 min, added to the seeded cells, incubated overnight, and replaced medium. The medium was harvested after 48 h and centrifuged at 4000 rpm at 4°C for 10 min. The supernatant containing the lentiviral vector was collected, filtered, aliquoted, and frozen at −80°C.

### Transduction

A549 cells were seeded at 300,000 cells in six-well plates and left overnight. Cells were infected with lentiviral vector for 24 h and followed by changing of culture medium. After another 48 h, the medium was changed to medium containing 6 µg/ml blasticidin (selective medium). Cells were cultured in selective medium and expanded into T-75 flask at day 7. After day 10, cells were maintained in medium supplemented with 4 µg/ml blasticidin. Cells were harvested at day 2 and 10 for RNA extraction and immunofluorescence assay (IFA).

### PCR array

Total RNA was isolated from A549 cells stably expressing MERS-N (test sample) or LacZ (control sample) proteins using RNeasy Mini kit (Qiagen) with genomic DNA (gDNA) removal with RNase-Free DNase set (Qiagen). RNAs were quantitated and used only when the absorbance ratio of OD_260 nm_/OD_280 nm_ was at least two. Total RNA of 3 µg was reverse transcribed into complementary DNA using the RT^2^ First Strand Kit (SA, Biosciences), mixed with the qPCR mastermix containing SYBR Green, and used on human antiviral response RT^2^ Profiler PCR arrays according to the manufacturer’s protocol (SA, Biosciences). The ABI StepOnePlus™ Real-Time PCR System was used to run the qPCR cycling program. The samples were repeated in two independent experiments. Then, *C*_T_ values were exported and analyzed by RT^2^ Profiler PCR Array data Analysis software version 4.

### IFA

A549 transduced cells were grown on coverslips. Approximately 24 h later, the medium was aspirated, and the cells were rinsed twice with PBS, fixed with 4% paraformaldehyde for 10 min, and permeabilized with 0.1% Triton X-100 for 10 min. Coverslips were then blocked in 1% bovine albumin serum for 30 min, and cells were incubated with mAb 7H6 for 1.5 h. After washing, cells were incubated with Alexa Fluor 488-conjugated goat anti-mouse IgG secondary antibodies (Invitrogen) for 1 h. After washing, cells were stained with DAPI before mounting. Images were captured with an Olympus FluoView FV1000 laser-scanning confocal microscope.

### Virus infection

Vero cells (ATCC) were seeded at 40,000 cells per well on a Nunc™ Lab-Tek™ Chamber Slide (ThermoFisher Scientific) in DMEM (Gibco) supplemented with 10% FBS (Gibco). After 24 h, cells were infected with MERS-CoV (Erasmus MC isolate) at a multiplicity of infection of 1 in DMEM for 1 h or mock infected. After 1 h, the cells were washed three times with PBS and followed by addition of DMEM with 10% FBS. After 48 h post-infection, the medium was removed. The cells were washed three times with PBS, fixed, and stained as described above. All virus work was performed in a biosafety level 3 (BSL-3) laboratory.

### Western blot analysis

Cell lysates were separated by SDS/PAGE, and proteins were transferred on to nitrocellulose membranes. The membranes were blocked with TBST (20 mM Tris [pH 7.5], 150 mM NaCl, and 0.1% Tween 20) containing 5% skim milk for 1 h. The blot was then incubated with primary antibody at 4°C overnight and washed thrice with TBST. This was followed by incubating with secondary antibody for 1 h, washed again with TBST, and finally developed through enhanced chemiluminescence. The primary antibodies used in the study included mAb 7H6 (as described above), anti-GAPDH polyclonal (Santa Cruz Biotechnology), and anti-FLAG polyclonal (Sigma) antibodies. Secondary antibodies used were HRP-conjugated goat anti-mouse and goat anti-rabbit IgG (Bio-Rad).

### Sandwich ELISA

The sandwich ELISA for CXCL10 (BD Biosciences) was used according to the manufacturer’s protocol.

### Statistical analysis

The unpaired two-tailed *t*-test was used to evaluate the significant differences of datasets obtained from at least three independent experiments. *P*<0.05 was considered statistically significant.

## Results and discussion

### MAb 7H6 is specific to MERS-N

**Figure 1:**
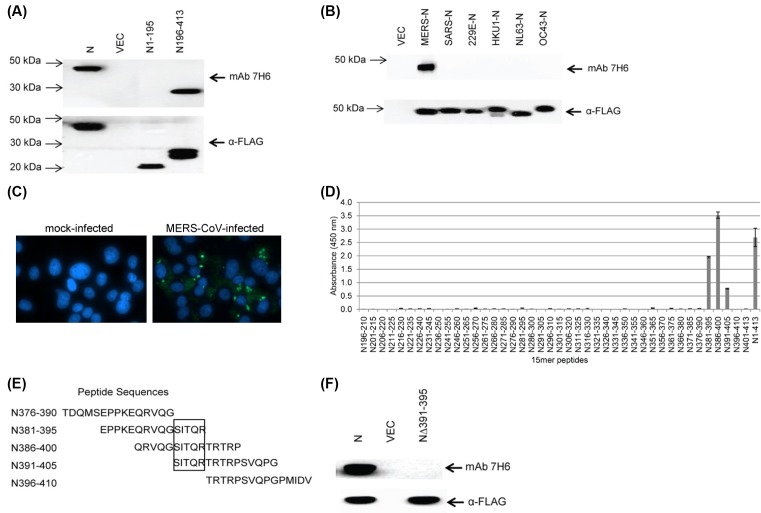
Characterization of mAb 7H6 (**A**) 293FT cells were transiently transfected with empty vector, FLAG-tagged full-length MERS-N, and its N- and C-terminal fragments. Western blot analysis was performed using mAb 7H6 and anti-FLAG antibody. (**B**) 293FT cells were transiently transfected with empty vector or FLAG-tagged N of different HCoVs. Western blot analysis was performed using mAb 7H6 or anti-FLAG antibody. (**C**) Vero E6 cells were mock infected or infected with MERS-CoV (multiplicity of infection of 1) and stained with mAb 7H6 at 2 days post-infection. (**D**) 15-mer peptides with ten amino acids overlapping sequences of the C-terminal fragment of MERS-N were generated and their binding to mAb 7H6 was analyzed by ELISA. Three independent experiments were performed and a representative data is shown. The results are mean values with error bars showing S.D. of triplicate wells. (**E**) Peptides between N376-410 were aligned and the minimal binding sequence was mapped (denoted in a box). (**F**) 293FT cells were transiently transfected with empty vector, FLAG-tagged MERS-N and mutant MERS-NΔ391-395. Western blot analysis was performed using mAb 7H6 and anti-FLAG antibody.

With the aim to generate an antibody that can be used to detect the expression of MERS-N protein in infected cells or transfected cells, a fragment corresponding to residues 196–413 in N was successfully expressed and purified from *E. coli* (data not shown). The purified protein was used to immunize mice and subsequently, mAb 7H6 (isotype of IgG2b) was produced. As shown in Figure 1A, mAb 7H6 bound to full-length MERS-N and the C-terminal fragment of MERS-N. As it would be expected, mAb 7H6 did not bind to the N-terminal fragment consisting of residues 1–195 in MERS-N. The human coronaviruses (HCoVs), including SARS, 229E, HKU1, NL63 and OC43, cause respiratory diseases. Therefore, there is a need to determine the cross-reactivity of mAb 7H6 to the N proteins of other HCoVs. As shown in Figure 1B, mAb 7H6 did not recognize N proteins of the other HCoVs indicating its specificity to the MERS-N protein.

Next, mAb 7H6 was used on MERS-CoV infected cells. As shown in Figure 1C, specific staining was observed in cells infected with MERS-CoV indicating that mAb 7H6 could detect MERS-N expressed during infection. MERS-N was observed to localize throughout the cytoplasm as well as in punctate cytoplasmic organelles. No staining was observed in mock-infected cells.

In order to determine the binding site for mAb 7H6, 15-mer peptides with ten overlapping amino acids from the C-terminal fragment of MERS-N were synthesized. Based on ELISA, mAb 7H6 bound significantly to peptides containing residues 381–395, 386–400, and 391–405 (Figure 1D), and alignment of these peptides indicates that residues 391–395 (SITQR) are essential for the interaction with mAb 7H6 (Figure 1E). Western blot analysis showed that mAb 7H6 did not bind to a mutant MERS-NΔ391-395aa, confirming that the SITQR motif is essential for binding (Figure 1F).

### Stable expression of the MERS-N protein regulated several antiviral response genes at the transcriptional level

**Figure 2:**
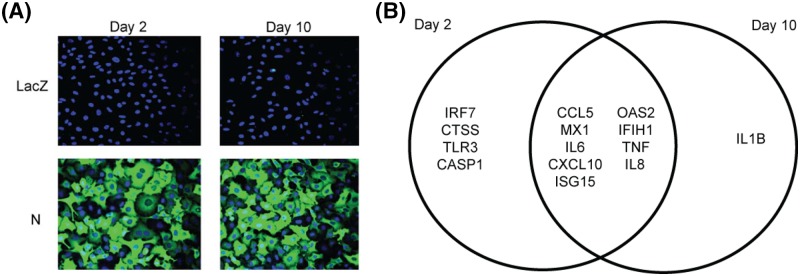
Selection of genes for downstream validation (**A**) A549 cells were transduced with lentiviral virus to express MERS-N and LacZ. Cells were subsequently selected in blasticidin, and protein expression was analyzed at day 2 and 10 post-selection by IFA using mAb 7H6. (**B**) RNA transcripts were obtained from A549-transduced cells expressing MERS-N or LacZ at day 2 and 10 post-selection and assayed using an 84-gene antiviral PCR array. Gene regulation was analyzed with a cutoff of 4-fold change and genes up-regulated on both day 2 and 10 post-selection are represented in a Venn diagram. The intersection shows common genes up-regulated on both days.

To determine if the MERS-N protein can modulate antiviral response genes, it was overexpressed in A549 cells using a lentiviral system. With the availability of mAb 7H6, N was expressed without any tag. The A549 transduced cells expressed relatively high MERS-N protein on both day 2 and 10 post-selection in an antibiotics medium as shown in Figure 2A. This suggests that MERS-N protein can be expressed and maintained for a period of time without cytotoxicity. At day 2 and 10 post-selection, RNA transcripts were obtained from transduced cells, and the expression of 84 human antiviral response genes were analyzed by RT-qPCR. Fold regulations of antiviral response genes by MERS-N protein was compared with negative control cells, which were expressing LacZ, by using the 2^−ΔΔ*C*^_T_ method. As shown in Supplementary Tables S1 and S2, several genes were found to be differentially regulated in the MERS-N expressing cells when compared with negative control cells.

A fold regulation cutoff of four was used to select for genes that were up- or down-regulated at both day 2 and 10. Nine genes, namely *CCL5, MX1, IL6, CXCL10, ISG15, OAS2, IFIH1, TNF* and *IL8*, were found to be up-regulated in both days as represented in the Venn diagram (Figure 2B). All of these genes with exception of IFIH1 maintained or had increases in fold regulation between day 2 and 10 suggesting that MERS-N persistently regulates these cytokines/chemokines (Supplementary Tables S1 and S2).

### Transient expression of MERS-N protein up-regulated CXCL10 at both transcriptional and translation levels

**Figure 3:**
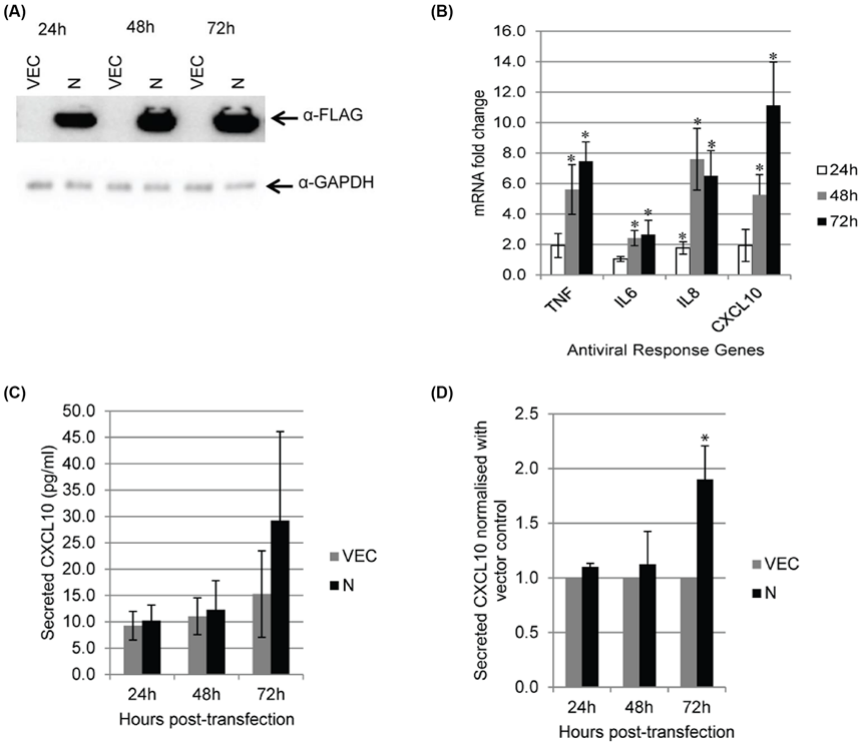
Expression of the four selected antiviral genes in 293FT cells (**A**) 293FT cells were transiently transfected with empty vector and FLAG-tagged MERS-N. Western blot analysis was performed using anti-FLAG antibody. (**B**) RNA transcripts from transiently transfected 293FT cells were assayed by RT-qPCR with TaqMan probes to analyze the mRNA fold changes of TNF, IL6, IL8, and CXCL10. mRNA expression of genes was first normalized against GAPDH and subsequently, MERS-N up-regulation was normalized against levels in vector-transfected cells. (**C**) Cell supernatants were harvested and CXCL10 secretion was evaluated using ELISA. (**D**) The secretion of CXCL10 by MERS-N was normalized against the vector. The results of these experiments were expressed as mean ± S.D. (error bars) of five independent experiments. Asterisk (*) indicates statistical significance of *P*<0.05 when compared with vector-transfected cells at the respective time-points.

A transient expression system was then used to express MERS-N so as to rule out host gene expression changes that may be caused by prolonged antibiotics selection. A highly transfectable cell 293FT was used to express FLAG-tagged or untagged MERS-N. As shown in Figure 3A, expression of FLAG-tagged MERS-N was detected at 24–72 h post-transfection, and the level of expression increased with time. Out of the nine host genes identified using stably transduced A549 cells, proinflammatory cytokine/chemokines TNF, IL6, IL8, and CXCL10 were reported to be up-regulated in MERS-CoV infected cells [3,17,20]. Hence, their levels in cells expressing FLAG-MERS-N were determined using RT-qPCR and normalized to vector-transfected cells. As shown in Figure 3B, all four genes were significantly up-regulated in cells expressing FLAG-MERS-N. Similar results were obtained when untagged MERS-N was expressed in 293FT (Supplementary Figure S1), suggesting that the FLAG tag at the N-terminal of MERS-N does not affect its function and can be used when analyzing mutants of MERS-N.

**Figure 4:**
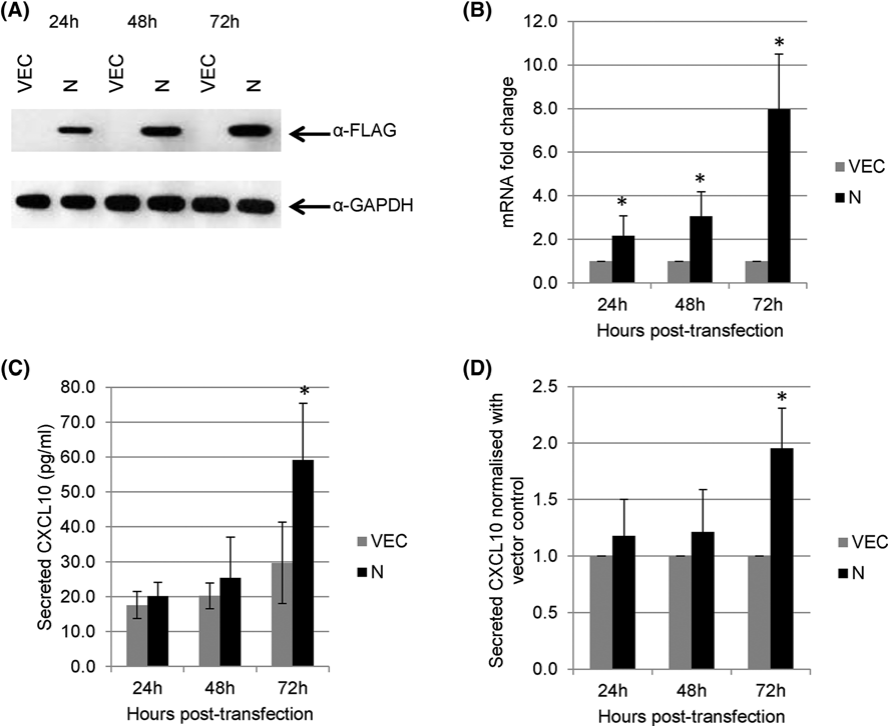
Expression of CXCL10 in A549 cells (**A**) A549 cells were transiently transfected with empty vector and FLAG-tagged MERS-N. Western blot analysis was performed using anti-FLAG antibody. (**B**) RNA transcripts from transiently transfected A549 cells were assayed by RT-qPCR with TaqMan probes to analyze the mRNA fold changes for the expression of CXCL10. mRNA expression of CXCL10 was first normalized against GAPDH and then normalized against levels in vector-transfected cells. (**C**) Cell supernatants were harvested from the transiently transfected cells, and CXCL10 secretion was evaluated using ELISA. (**D**) The secretion of CXCL10 of cells overexpressing MERS-N was normalized against the vector. The results of these experiments were expressed as mean ± S.D. (error bars) of six independent experiments in triplicates. The asterisk (*) indicates statistical significance of *P*<0.05 when MERS-N is compared with vector control.

Next, the secretions of these cytokines/chemokines into the culture supernatant were measured. Secretions of CXCL10 were observed in MERS-N expressing cells at 72 h post-transfection when compared with vector-transfected cells (Figure 3C). Although CXCL10 secretion from MERS-N expressing cells was relatively low, a consistent increase of approximately 2-fold was observed in different experiments when normalized with the vector-transfected cells (Figure 3D). Transient transfection of A549 cells was also performed and high expression of MERS-N was achieved (Figure 4A). Similarly to the observation in 293FT cells, the overexpression of MERS-N in A549 cells resulted in a significant increase in the mRNA levels of CXCL10 (Figure 4B) and its secretion (Figure 4C,D) when compared with vector-transfected cells. This suggests that MERS-N protein regulates both the transcriptional and translational expression of CXCL10. In contrast, TNF, IL6, and IL8 secretions from MERS-N expressing cells were not significantly up-regulated when compared with the vector-transfected cells (data not shown).

### A fragment consisting of residues 196–413 in MERS-N is sufficient to up-regulate CXCL10

**Figure 5:**
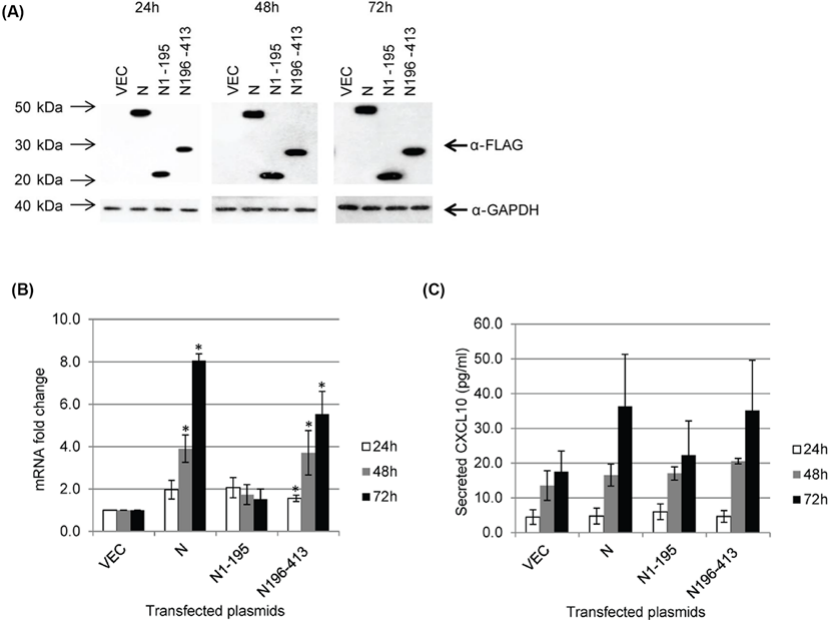
MERS-N regulates CXCL10 through its C-terminal fragment (**A**) 293FT cells were transiently transfected with empty vector, FLAG-tagged N, N1-195, and N196-413. Western blot analysis was performed using anti-FLAG and anti-GAPDH antibodies. (**B**) RNA transcripts were obtained from transiently transfected 293FT cells and assayed by RT-qPCR to analyze the mRNA fold changes of CXCL10. (**C**) Cell supernatants were harvested from transiently transfected 293FT cells, and CXCL10 secretion was evaluated using ELISA. The results of these experiments were expressed as mean ± S.D. (error bars) of three independent experiments in triplicates. Asterisk (*) indicates statistical significance of *P*<0.05 when compared with vector-transfected cells at the respective time-points.

Based on alignment with the N proteins of other CoVs, MERS-N is proposed to be organized into two main folded domains, which are likely to be independent of each other, separated by a linker region that contains a SR-rich motif [12]. The N-terminal domain consists of residues 37–164, while the C-terminal domain consists of residues 239–362. In addition, residues 1–36 and 363–413 are classified as intrinsically disordered regions. Here, two fragments of MERS-N, namely N1-195 and N196-413, were constructed and found to be expressed at similar levels as full-length N when transfected into 293FT cells (Figure 5A). As shown in Figure 5B, the N196-413 fragment was able to up-regulate CXCL10 transcriptionally, while the N1-195 did not have a significant effect. Consistently, both full-length N and N196-413, but not N1-195, increased the secretion of CXCL10 when compared with vector-transfected cells (Figure 5C). As N1-195 contains N-terminal domain and SR-rich motif, it appears that these domains are not involved in the up-regulation of CXCL10. N196-413 contains part of the linker region, C-terminal domain and the intrinsically disordered region at the C-terminal tail and further studies are required to determine which of these domains are required for up-regulation CXCL10 expression.

## Summary

MERS-CoV has broad tissue tropism including lower airway, intestinal tract, liver, kidney, neuronal, monocyte, and T-lymphocyte cells [5,22]. Upon infection by MERS-CoV, cytokine/chemokine regulations have been reported in airway epithelial cells and lung fibroblast cells [17] as well as monocyte-derived macrophages and dendritic cells [3,20,23]. In the present study, we showed that the overexpression of MERS-N protein in A549 (lung) and 293FT (kidney) up-regulated TNF, IL6, IL8, and CXCL10 on the transcriptional levels, suggesting that MERS-N protein may be able to regulate multiple antiviral genes in different tissues. On the protein level, the overexpression of MERS-N protein in 293FT also increased the secretion of CXCL10, albeit at low levels, but not TNF, IL6, and IL8. It is not clear that why the secretion of CXCL10 was low despite a robust up-regulation on mRNA levels by approximately 10-fold in MERS-N expressing cells, but one possible reason could be the low efficiency of cytokine/chemokine secretion from 293FT cells. Similarly, the overexpression of MERS-N protein in A549 cells also led to transcriptional and translational up-regulation of CXCL10. As MERS-CoV has been reported to infect immune cells, further studies could be performed to study the effects of MERS-N on the expression of antiviral genes in different types of immune cells.

CXCL10 is a cytokine belonging to the CXC chemokine family and binds to the CXCR3 receptor to induce chemotaxis, apoptosis, cell growth, and angiostasis. Expression levels of CXCL10 have been associated with inflammatory diseases including infectious diseases, immune dysfunction, and tumor development [19,24]. High expressions of CXCL10 have been reported in individuals infected with pathogens including viruses, bacteria, fungi, and parasites [24]. CXCL10 regulation has been reported in MERS patients [18,19] as well as MERS-CoV infected cells [3,17,20]. Our results suggest that the MERS-N protein is one of the contributing factors for CXCL10 up-regulation during infection. In addition, a fragment of MERS-N consisting of residues 196–413 is sufficient to up-regulate CXCL10. Future works will focus on identifying the exact motif in MERS-N that is contributing to the up-regulation of CXCL10, and delineating the relative contribution of MERS-N and different viral proteins to this up-regulation which may have implication for viral pathogenesis.

## Supporting information

**Supplementary Figure S1:** Regulation of host gene expression by FLAG-tagged MERS-N compared to untagged MERS-N. mRNA transcripts were obtained from 293FT cells transiently transfected with FLAG-tagged MERS-N or untagged MERS-N plasmids for 24h to 72h. RT-qPCR was performed to evaluate the mRNA expression of (A) TNF, (B) IL6, (C) IL8 and (D) CXCL10. Results are means of fold changes with error bars showing standard deviation (SD) of values from triplicate wells.

**Supplementary Table 1:** Regulation of antiviral response genes by MERS-N protein at day 2 post-selection (2 independent experiments).

**Supplementary Table 2:** Regulation of antiviral response genes by MERS-N protein at day 10 post-selection (2 independent experiments)

## Abbreviations

GST: glutathione S-transferase
CXCL10: C-X-C motif chemokine 10
IL6: interleukin-6
IL8: interleukin-8
HCoV: human coronavirus
IFA: immunofluorescence assay
MAb: monoclonal antibody
MERS: Middle East respiratory syndrome
MERS-CoV: MERS-coronavirus
N: nucleocapsid
RT-qPCR: reverse transcription-quantitative PCR
SARS: severe acute respiratory syndrome
SR: serine-arginine
TNF: tumor necrosis factor

## References

[1] WHO. MERS-CoV Fact Sheet, http://www.who.int/mediacentre/factsheets/mers-cov/en/ (date of access 18 June 2018)

[2] Channappanavar R. and Perlman S. (2017) Pathogenic human coronavirus infections: causes and consequences of cytokine storm and immunopathology. Semin Immunopathol 39, 529–539 10.1007/s00281-017-0629-x 28466096 PMC7079893

[3] Zhou J., Chu H., Li C. (2014) Active replication of Middle East respiratory syndrome coronavirus and aberrant induction of inflammatory cytokines and chemokines in human macrophages: implications for pathogenesis. J. Infect. Dis. 209, 1331–1342 10.1093/infdis/jit504 24065148 PMC7107356

[4] van den Brand J.M., Smits S.L. and Haagmans B.L. (2015) Pathogenesis of Middle East respiratory syndrome coronavirus. J. Pathol. 235, 175–184 10.1002/path.4458 25294366 PMC7167882

[5] Zhou J., Chu H., Chan J.F. (2015) Middle East respiratory syndrome coronavirus infection: virus-host cell interactions and implications on pathogenesis. Virol. J. 12, 218 10.1186/s12985-015-0446-6 26690369 PMC4687146

[6] Zaki A.M., van Boheemen S., Bestebroer T.M. (2012) Isolation of a novel coronavirus from a man with pneumonia in Saudi Arabia. N. Engl. J. Med. 367, 1814–1820 10.1056/NEJMoa1211721 23075143

[7] Chan J.F., Lau S.K., To K.K. (2015) Middle East respiratory syndrome coronavirus: another zoonotic betacoronavirus causing SARS-like disease. Clin. Microbiol. Rev. 28, 465–522 10.1128/CMR.00102-14 25810418 PMC4402954

[8] Verheije M.H., Hagemeijer M.C., Ulasli M. (2010) The coronavirus nucleocapsid protein is dynamically associated with the replication-transcription complexes. J. Virol. 84, 11575–11579 10.1128/JVI.00569-10 20739524 PMC2953146

[9] Lin S.Y., Liu C.L., Chang Y.M. (2014) Structural basis for the identification of the N-terminal domain of coronavirus nucleocapsid protein as an antiviral target. J. Med. Chem. 57, 2247–2257 10.1021/jm500089r 24564608 PMC3983370

[10] McBride R., van Zyl M. and Fielding B.C. (2014) The coronavirus nucleocapsid is a multifunctional protein. Viruses 6, 2991–3018 10.3390/v6082991 25105276 PMC4147684

[11] Ding B., Qin Y. and Chen M. (2016) Nucleocapsid proteins: roles beyond viral RNA packaging. Wiley Interdiscip. Rev. RNA, 7, 213–226 10.1002/wrna.1326 26749541 PMC7169677

[12] Papageorgiou N., Lichiere J., Baklouti A. (2016) Structural characterization of the N-terminal part of the MERS-CoV nucleocapsid by X-ray diffraction and small-angle X-ray scattering. Acta Crystallogr. D Struct. Biol. 72, 192–202 10.1107/S2059798315024328 26894667 PMC7159594

[13] Grunewald M.E., Fehr A.R., Athmer J. (2018) The coronavirus nucleocapsid protein is ADP-ribosylated. Virology 517, 62–68 10.1016/j.virol.2017.11.020 29199039 PMC5871557

[14] Cui L., Wang H., Ji Y. (2015) The nucleocapsid protein of coronaviruses acts as a viral suppressor of RNA silencing in mammalian cells. J. Virol. 89, 9029–9043 10.1128/JVI.01331-15 26085159 PMC4524063

[15] Hsin W.C., Chang C.H., Chang C.Y. (2018) Nucleocapsid protein-dependent assembly of the RNA packaging signal of Middle East respiratory syndrome coronavirus. J. Biomed. Sci. 25, 47 10.1186/s12929-018-0449-x 29793506 PMC5966903

[16] Mo Y. and Fisher D. (2016) A review of treatment modalities for Middle East respiratory syndrome. J. Antimicrob. Chemother. 71, 3340–3350 10.1093/jac/dkw338 27585965 PMC7109760

[17] Lau S.K., Lau C.C., Chan K.H. (2013) Delayed induction of proinflammatory cytokines and suppression of innate antiviral response by the novel Middle East respiratory syndrome coronavirus: implications for pathogenesis and treatment. J. Gen. Virol. 94, 2679–2690 10.1099/vir.0.055533-0 24077366

[18] Faure E., Poissy J., Goffard A. (2014) Distinct immune response in two MERS-CoV-infected patients: can we go from bench to bedside? PLoS ONE 9, e88716 10.1371/journal.pone.0088716 24551142 PMC3925152

[19] Kim E.S., Choe P.G., Park W.B. (2016) Clinical progression and cytokine profiles of Middle East respiratory syndrome coronavirus infection. J. Korean Med. Sci. 31, 1717–1725 10.3346/jkms.2016.31.11.1717 27709848 PMC5056202

[20] Chu H., Zhou J., Wong B.H. (2014) Productive replication of Middle East respiratory syndrome coronavirus in monocyte-derived dendritic cells modulates innate immune response. Virology 454–455, 197–205 10.1016/j.virol.2014.02.018 24725946 PMC7111975

[21] Oh H.L., Akerstrom S., Shen S. (2010) An antibody against a novel and conserved epitope in the hemagglutinin 1 subunit neutralizes numerous H5N1 influenza viruses. J. Virol. 84, 8275–8286 10.1128/JVI.02593-09 20519402 PMC2916527

[22] Chan J.F., Chan K.H., Choi G.K. (2013) Differential cell line susceptibility to the emerging novel human betacoronavirus 2c EMC/2012: implications for disease pathogenesis and clinical manifestation. J. Infect. Dis. 207, 1743–1752 10.1093/infdis/jit123 23532101 PMC7107374

[23] Tynell J., Westenius V., Ronkko E. (2016) Middle East respiratory syndrome coronavirus shows poor replication but significant induction of antiviral responses in human monocyte-derived macrophages and dendritic cells. J. Gen. Virol. 97, 344–355 10.1099/jgv.0.000351 26602089 PMC4804640

[24] Liu M., Guo S., Hibbert J.M. (2011) CXCL10/IP-10 in infectious diseases pathogenesis and potential therapeutic implications. Cytokine Growth Factor Rev. 22, 121–130 21802343 10.1016/j.cytogfr.2011.06.001 PMC3203691

