# Supplementary material for: Overexpression of the nucleocapsid protein of Middle East respiratory syndrome coronavirus up-regulates CXCL10
Source: Biosci Rep. 2018 Oct 17;38(5):BSR20181059. doi: 10.1042/BSR20181059 (PMC6200698; doi:10.1042/BSR20181059)
Supplement: Supplementary file 1 [file bsr20181059_Supp1.pdf]

Supplementary data

Overexpression of the nucleocapsid protein of Middle East Respiratory Syndrome coronavirus up-regulates CXCL10

James Odame Aboagye[1,2], Chow Wenn Yew[1], Oi-Wing Ng[2],
Vanessa M. Monteil[3,4], Ali Mirazimi[3,4], Yee-Joo Tan[1,2,*]

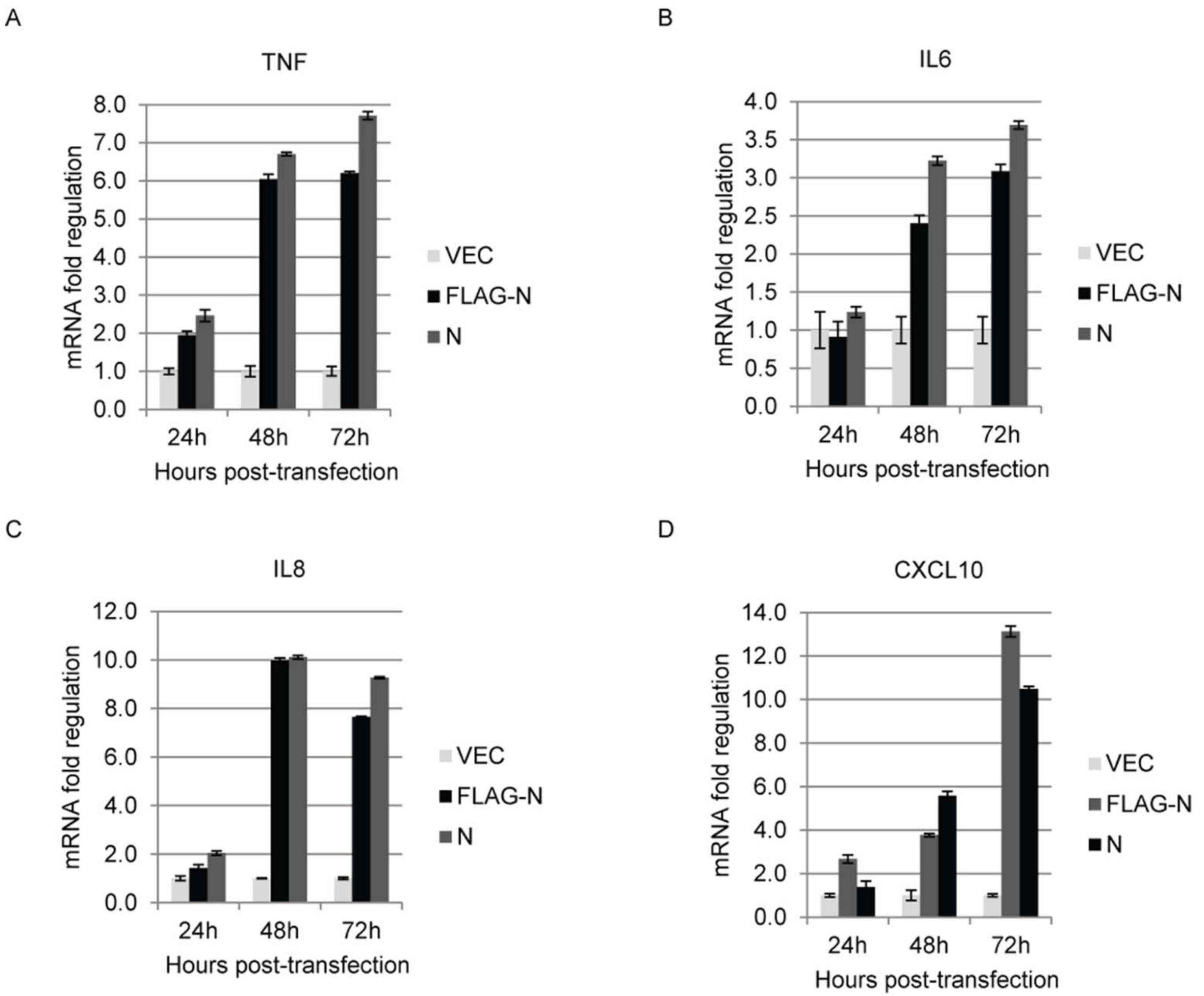

Supplementary Figure S1: Regulation of host gene expression by FLAG-tagged MERS-N compared to untagged MERS-N. mRNA transcripts were obtained from 293FT cells transiently transfected with FLAG-tagged MERS-N or untagged MERS-N plasmids for 24h to 72h. RT-qPCR was performed to evaluate the mRNA expression of (A) TNF, (B) IL6, (C) IL8 and (D) CXCL10. Results are means of fold changes with error bars showing standard deviation (SD) of values from triplicate wells.

Supplementary Table 1: Regulation of antiviral response genes by MERS-N protein at day 2 post-selection (2 independent experiments).

No.	Gene Symbol	Fold Change (Mean)	Fold Change (1) MERS-N/LacZ	Fold Change (2) MERS-N/LacZ	Std Deviation
1	CCL5	12.92	13.84	11.99	1.31
2	MX1	12.83	12.92	12.73	0.13
3	IRF7	10.61	11.17	10.06	0.79
4	IL6	9.49	9.76	9.23	0.38
5	CXCL10	8.53	9.74	7.32	1.71
6	ISG15	8.13	8.96	7.29	1.18
7	CTSS	7.57	7.96	7.18	0.55
8	TLR3	7.48	7.87	7.08	0.56
9	OAS2	6.97	7.30	6.65	0.46
10	IFIH1	6.04	5.46	6.62	0.82
11	CASP1	5.95	6.27	5.64	0.45
12	TNF	5.89	4.89	6.89	1.42
13	CXCL8	5.63	5.87	5.39	0.33
14	IFNA2	3.64	3.60	3.69	0.06
15	IFNB1	3.53	3.63	3.44	0.13
16	PSTPIP1	3.48	4.41	2.55	1.32
17	TRIM25	3.28	3.77	2.79	0.69
18	CYLD	3.21	3.59	2.83	0.54
19	DDX58	3.05	3.21	2.89	0.23
20	IL12A	2.85	2.90	2.80	0.07
21	CTSB	2.86	3.24	2.49	0.53
22	NLRP3	2.85	3.29	2.40	0.63
23	SPP1	2.74	2.86	2.61	0.18
24	STAT1	2.71	3.06	2.35	0.50
25	AIM2	2.79	3.78	1.80	1.40
26	CARD9	2.60	2.78	2.42	0.26
27	IRF5	2.58	3.02	2.14	0.62
28	PYCARD	2.58	3.06	2.10	0.68
29	IFNA1	2.44	2.57	2.31	0.18
30	IFNAR1	2.44	2.75	2.12	0.45
31	TRADD	2.36	2.44	2.28	0.11
32	TLR7	2.33	2.60	2.05	0.39
33	AZI2	2.25	2.38	2.12	0.18
34	CTSL	2.18	2.26	2.10	0.12
35	CASP10	2.18	2.47	1.89	0.41
36	IKBKB	2.15	2.28	2.02	0.18
37	ATG5	2.16	2.44	1.88	0.40
38	IL1B	2.11	2.07	2.14	0.05
39	NFKB1	2.11	2.28	1.93	0.25
40	FOS	2.08	2.28	1.87	0.29
41	IL12B	2.14	1.60	2.68	0.76
42	IL15	2.04	2.11	1.97	0.10

Supplementary Table 2: Regulation of antiviral response genes by MERS-N protein at day 10 post-selection (2 independent experiments)

No.	Gene Symbol	Fold Change (Mean)	Fold Change (1) MERS-N/LacZ	Fold Change (2) MERS-N/LacZ	Std Deviation
1	CXCL10	86.52	53.44	119.60	46.78
2	CCL5	55.78	58.01	53.55	3.16
3	TNF	28.38	35.00	21.75	9.37
4	OAS2	25.79	32.08	19.49	8.90
5	MX1	14.15	14.40	13.91	0.35
6	IL6	9.47	9.34	9.61	0.19
7	ISG15	9.22	8.58	9.86	0.91
8	IL1B	6.25	5.71	6.80	0.77
9	CXCL8	5.97	5.82	6.12	0.21
10	IFIH1	5.35	5.13	5.57	0.31
11	CTSS	3.40	3.30	3.51	0.15
12	IRF7	3.39	3.22	3.56	0.24
13	DDX58	2.61	2.21	3.01	0.57
14	CASP1	2.52	2.59	2.45	0.10
15	IL15	2.21	2.13	2.30	0.12
16	CD40	2.05	1.85	2.24	0.28
17	PSTPIP1	-2.11	-2.73	-1.49	0.88
18	CXCL9	-2.16	-1.99	-2.33	0.24
19	PYDC1	-2.70	-2.83	-2.57	0.18
